# Developing a Decision-Making Model for Construction Safety Behavior Supervision: An Evolutionary Game Theory-*Based* Analysis

**DOI:** 10.3389/fpsyg.2022.861828

**Published:** 2022-04-07

**Authors:** Xin Ning, Yu Qiu, Chunlin Wu, Kexin Jia

**Affiliations:** ^1^School of Investment and Construction Management, Dongbei University of Finance and Economics, Dalian, China; ^2^School of Economics and Management, Beihang University, Beijing, China; ^3^Beijing Key Laboratory of Emergency Support Simulation Technologies for City Operations, Beihang University, Beijing, China

**Keywords:** construction safety supervision, decision-making model, the enterprise entity responsibility mechanism, the third-party participation mechanism, evolutionary game theory

## Abstract

Without the active participation of enterprises and front-line workers, it is difficult for the government to perform effective supervision to ensure behavioral safety among front-line workers. To overcome inadequate government supervision and information attenuation caused by vertical management mode and limited resources, and to change passive supervision into active control with the proactive participation of enterprises and workers, this paper combines the entity responsibility mechanism and the third-party participation mechanism based on government supervision to analyze the decision-making process of government and enterprises on safety behavior supervision. An evolutionary game model was established to describe the decision-making interactions between the government and construction enterprises under the two mechanisms, and a simulation was performed to illustrate the factors influencing the implementation of the mechanisms. The results show that both mechanisms have a positive effect on government supervision, and the third-party participation mechanism was found to be working better. The implementation of the two mechanisms is influenced by punishment, subsidy, and cost, and it has different sensitivities to the three influencing factors. This study provides a theoretical framework for enhancing the government supervision mechanism, and the decision-making between the government and construction enterprises enhances the management form and guides their actual supervision practices.

## Introduction

Accidents in the construction industry have been a serious global issue for a long time; moreover, industrial safety is a matter of utmost significance (Jin et al., 2020; Li Z. et al., 2021). The government, as an external constraint force, has been recognized as critical for construction safety (Zeng and Chen, 2015), however, the statistics on industrial accident casualties indicate that there are still some defects in government supervision. On the one hand, a lack of personnel and resources results in inadequate government supervision (Cao and Du, 2018; Gong et al., 2021). On the other hand, China’s safety supervision system adopts vertical management, which entails that government supervision of construction safety is unified management and hierarchical responsibility (Ma and Zhao, 2018). Under these circumstances, government supervision is limited by the attenuation of the level-by-level effect, such that the unsafe behavior of front-line workers, which is the primary and immediate cause of accidents, cannot be controlled at a fundamental level (Blackmon and Gramopadhye, 1995; Li et al., 2017; Yu et al., 2017). Construction enterprises are the main decision-makers when it comes to safe production. They can directly supervise front-line workers, and the government can mobilize enterprises to jointly participate in the mission of safe production and safety supervision to improve the supervision of front-line workers (Bu, 2016a). Furthermore, when there is an excess workload, enterprises can employ a third party, which specializes in supervision, to control the unsafe behavior of employees, thus avoiding the lack of professional supervision of enterprises and sharing the responsibility. Therefore, based on government supervision, it is imperative to explore a more proactive and professional supervision pathway that can realize active control before an accident occurs and strengthen the efficiency of government supervision.

Previous studies have focused on passive supervision mechanisms, including safety-related regulations and information systems, to control the unsafe behavior of front-line workers (Koh and Rowlinson, 2012; Pi et al., 2019; Fang et al., 2020). However, to promote effective supervision, previous research mostly optimizes the supervision mechanism from the perspective of the government (Guo et al., 2018), lacks the combination of the government’s passive safety supervision and enterprises’ active safety supervision, and does not emphasize the decision-making process between the government and enterprises. However, from the government’s perspective, regardless of the measures implemented, the hierarchical supervision mode of the government cannot be changed. Information asymmetry, which leads to the behavior of the liability subject, cannot be effectively restrained. Therefore, decision-making interactions between the government and enterprises in safety supervision must be analyzed.

To solve the problems of information attenuation and inadequate government supervision, we explored a safety supervision mode that implements the enterprise entity responsibility mechanism (Lu, 2020) and third-party participation mechanism (Zhu and Chen, 2015; Li X. C. et al., 2021) based on the government supervision. The enterprise entity responsibility mechanism means that the enterprise is the main body of responsibility for production safety, and the chief person in charge is the first person responsible for production safety, who takes the initiative to fulfill the work safety responsibility. The third-party participation mechanism refers to enterprises implementing a third-party safety supervision organization that is independent of the government or construction enterprises to provide professional, objective, and fair safety supervision services for the construction enterprises. Differences in interests and objectives lead to construction safety supervision under the two mechanisms of a game process between government and construction enterprises (Bu, 2016a). To better examine the dynamic change process of construction safety supervision, this study introduces the evolutionary game theory to analyze the behaviors of different subjects. Simultaneously, the punishment, subsidy, and probability of accidents under different supervision mechanisms are considered in the game model, which makes the research more realistic.

In contrast to previous studies, herein, we focus on the choice of supervision mechanism in different scenarios from the perspective of enterprises and their influence on government supervision decision-making. Therefore, to verify the effectiveness and to promote the implementation of the two abovementioned mechanisms for government safety supervision, the decision-making interactions of government and construction enterprises were quantitatively analyzed to ascertain the behavioral characteristics. Furthermore, in this study, the equilibrium points of the game system were simulated to validate and compare the two mechanisms to prove the effectiveness of supervision. Corresponding suggestions were put forward on the degree of government subsidies and punishments for construction enterprises according to the analysis results. This study presents the realistic supervision process under two mechanisms. The model describes the decision-making interactions between construction enterprises and the government and reveals the inherent law in the supervision process, which not only enhances the supervision mechanism, but also contributes to government policy formulation and enterprise supervision.

## Literature Review

### Construction Safety Supervision

Safety supervision refers to the comprehensive supervision and inspection of the safety conditions and implementation of the safety responsibilities of the relevant subjects by the construction administrative departments and relevant government departments in accordance with laws, regulations, and relevant standards (Cheng et al., 2013). The problems of the hierarchical supervision mode in China have facilitated extensive studies on designing effective safety incentive mechanisms and led to changes in the passive situations of construction safety to realize active control, that is, implementation of certain measures that urge the enterprises to give more emphasis to the safety management and form an enterprise-based self-operation mechanism of safety management. For example, it has been proposed that successful safety supervision largely depends on employee involvement, as workers tend to support the activities that they themselves help to create (Aksorn and Hadikusumo, 2008). Next, some research focused on the structure of responsibility, incentives, and penalties in the enterprises, which reencourage positivity and creativity among workers, resulting in conscious observation of all kinds of safety rules and regulations for production and accomplishment of the goal (Liang, 2006; Ji et al., 2021). The relationship between project safety performance and the influence of construction enterprises has been examined (Hallowell, 2010). Owners are making efforts to improve project safety performance, with a focus on achieving the goal of zero injuries, selecting safe contractors, and developing a safety culture on their projects through safety training and safety recognition programs (Huang and Hinze, 2006). A double closed-loop feedback control system based on the security problems of development and construction units has been proposed to promote the efficiency of safety supervision (Bu, 2016b). In addition, a type of engineering construction safety consultant model has been proposed. In other words, a professional agency is entrusted with carrying out on-site safety management. Professional safety engineers take full advantage of their knowledge and experience and they have significantly improved the management level of development organizations (Liu et al., 2013).

In general, the above research proves that the participation of enterprises, employees, and third parties can strengthen the effectiveness of government supervision. However, most existing studies focus only on the effect of an enterprises’ active supervision on safety supervision performance. As the government is the main body of supervision, government supervision is indispensable. The following factors need to be explored: (1) The effect of active supervision of enterprises on the decision-making of government supervision. (2) Ways to coordinate both active and government supervision to achieve optimal decision-making strategies. It is a game process between enterprises’ active supervision and the government’s passive supervision; therefore, it is necessary to abstract the practical problems of supervision in the game model and study the interactive decision-making between them to promote active control and improve the efficiency of government supervision.

### Evolutionary Game Theory in Construction Safety Supervision

To better examine the process of safety supervision, the evolutionary game theory was introduced to analyze the behaviors of different subjects. Compared to the classical game theory, the evolutionary game theory is a combination of game theory and dynamic evolution process analyses, and it focuses more on the dynamics of strategy change (Weibull, 1997). Supervision is a controllable process, and the study of evolutionary games is beneficial to excavate the decision logic behind the behaviors of stakeholders and to reduce or even avoid accidents at the root (Tang et al., 2021).

To analyze the key factors of a stable construction safety supervision, an evolutionary game model has been presented, which demonstrates the need to introduce an appropriate external supervision and restraint mechanism that enhances both sides to control safety risk (Zeng and Chen, 2015). Some scholars have established a game model of government departments, as well as upstream and downstream participants to examine the effectiveness of China’s construction project quality supervision system and proposed a dynamic punishment and incentive method (Feng et al., 2020). In addition, the behavioral strategy choices and the change in the stable state of the production staff and safety supervisor under different scenarios are discussed. The results showed that the stable state of unsafe employee behavior supervision was not related to profit (Shi et al., 2018). Other scholars have analyzed the interaction of project owners, supervision engineers, and construction contractors in construction quality supervision and verified that the dynamic reward and punishment mechanism can improve the quality of the supervision procedure (Guo et al., 2018).

In summary, the evolutionary game theory approach has been gradually introduced in various studies to analyze construction safety supervision behaviors in engineering projects. However, most existing studies have focused on illustrating the game process between the main participants in supervision and safety production; however, the reports are not convincing enough to regard construction enterprises as active supervision participants and explore decision-making interactions with the government. At the same time, the existing research lacks a comparison of the different active supervision mechanisms. Therefore, an evolutionary game model between the government and construction enterprises is formulated, and the decision-making interactions are described. The impact factors of implementing the two mechanisms by enterprises in different scenarios are analyzed and compared to provide constructive suggestions for the enthusiasm of enterprises and optimizing safety supervision mechanisms.

## Construction Safety Supervision Decision-Making Model

**FIGURE 1:**
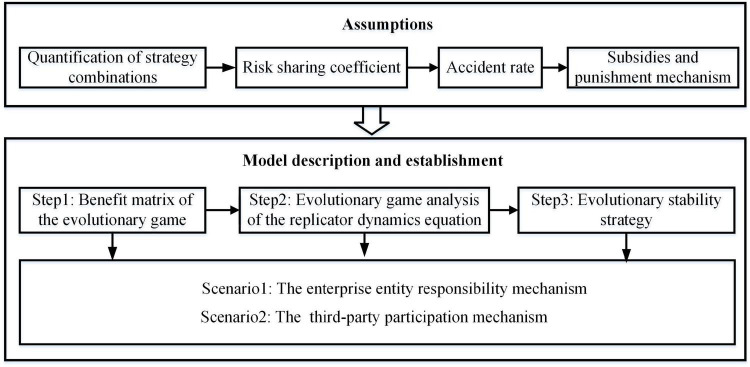
The game analysis process of construction safety supervision.

In this study, two supervision mechanisms, i.e., the enterprise entity responsibility and the third-party participation mechanisms, are added to the game model between construction enterprises and the government. At the same time, the decision paths and stable strategies are demonstrated, in which the influence of the bounded rationality of both sides is considered. The game analysis process for construction safety supervision is illustrated in Figure 1.

### Assumptions

To analyze the safety supervision in the construction field, we made the following assumptions under the two supervision mechanisms.

**Assumption 1:** The game has two participants, namely the government and construction enterprises, and both parties have two strategies to choose from. The government performs supervision over the construction operations to (1) avoid adverse effects from accidents, and (2) provide a cost-effective service of real-time monitoring of all construction enterprises under jurisdiction, since it is quite expensive; thus, they will choose the strength of safety supervision. Therefore, the government’s strategy is {strict supervision *G*_*i*1_, ordinary supervision *G*_*i*2_}. (*i* = 1 and *i* = 2 represent the two mechanisms). Ordinary supervision entails that, based on completing policy formulation and rulemaking, the government will conduct random inspections of enterprises according to the probability of β. While on one hand, strict supervision means that in addition to ordinary safety supervision, the scope of supervision is enhanced; on the other hand, punishment is increased, and the safety behavior of enterprises is more strictly supervised. Moreover, the behavioral strategy set of the construction enterprises is {implementing the mechanism *C*_*i*1_, not implementing the mechanism *C*_*i*2_}. The probabilities of the construction enterprises choosing the “implementing the mechanism” or “not implementing the mechanism” strategies are *x* and 1*-x*, respectively. The probabilities of the government choosing the “strict supervision” or “ordinary supervision” strategies are *y*and 1-*y*, respectively.

**Assumption 2**: Referring to Yu et al. (2020) and Meng et al. (2021), the participants in the game are required to share the loss of the liability cost reasonably, and the loss of the liability cost is linearly related (the correlation coefficient is a real number greater than 0). That is, if construction enterprises bear a liability cost loss of *C*, then the government bears a liability cost loss of *kC*, where *k* is the transfer coefficient of the liability cost loss.

**Assumption 3**: According to He et al. (2020) and Wu et al. (2020), the capabilities of both players have complementary effects. In other words, when the construction enterprises implement the mechanism under strict government supervision, the possibility of accidents is minimal. Without loss of generality, we assume that the accident cost loss is zero in this case. When one side chooses ordinary safety management while the other side chooses to implement the mechanism, the probability of accidents increases. When both sides adopt opposing safety management strategies, the probability of risk loss is the greatest, we assume that an accident must occur.

**Assumption 4**: According to the “Production Safety Law of the People’s Republic of China,” the state will reward units and individuals that have made remarkable achievements in improving production safety conditions, preventing production safety accidents, and participating in emergency rescue. It also clearly stipulates that the violations of safety production found during the inspection shall be corrected on the spot or corrected within a specified time limit. For acts that should be given administrative penalties according to the law, administrative penalty decisions shall be made. We assumed that no matter what the government chooses between strict supervision and ordinary supervision, subsidies will be given to enterprises that implement the two mechanisms. When enterprises do not implement the two mechanisms and the government performs strict supervision, enterprises will be punished. When the government chooses ordinary supervision, since it only completes the most basic policy formulation and supervision tasks, the intensity of supervision and punishment is small, so the cost of punishment is ignored under this circumstance. Based on the above assumptions, we established a game model for the government and enterprises under the two mechanisms, i.e., the entity responsibility mechanism and the third-party participation mechanism.

### Model Description and Establishment

#### Scenario 1: The Enterprise Entity Responsibility Mechanism

***Step 1.*** *Benefit matrix of the evolutionary game*

Under the enterprise entity responsibility mechanism, the construction enterprises undertake the most direct and important responsibility in the construction process, which entails those enterprises are willing to invest more in safety supervision and actively carry out safety management to avoid safety accidents. When the probability of safety accidents is reduced, the government can get rewards from superior departments and praise from the public, and the shared cost of the accidents is also reduced. Therefore, for construction enterprises, the cost, and subsidies for implementing the mechanism are *C*_*c*1_ and *S*_1_, respectively. If construction enterprises fail to fulfill the entity responsibility, the loss caused by the accidents is *L*_1_, and they will be fined *F*_1_ by the government. For the government, when it adopts strict supervision, the work cost is *C*_*g*1_ and the benefits from superior departments and increased public credibility are *R*_1_. In contrast, when the government chooses ordinary supervision, the work cost is β*C*_*g*1_ (0 < β < 1), and it will lose reputation *M*_1_ if an accident occurs. When enterprises implement the mechanism and the government chooses ordinary supervision, the probability of accidents is *h* (0 < *h* < 1); when enterprises do not use the mechanism and the government chooses strict supervision, the probability of accidents is *g* (0 < *g* < 1). Because enterprises can share part of the government’s supervision tasks, it is assumed that *g* > *h.* Finally, the transfer coefficient of the liability cost loss by the government is *k*.

According to these assumptions, the benefit matrix of the government and construction enterprises under enterprise entity responsibility can be constructed, as shown in Table 1.

***Step 2.*** *Evolutionary game analysis of the replicator dynamics equation*

**TABLE 1:** The benefit matrix between government and construction enterprises.

Construction enterprises	Government	
	Strict supervision (*y*)	Ordinary supervision (1-*year*)
Implementing the mechanism(*x*)	*–Cc*_1_ *+S*_1_ *,R1-C_*g*_*_1_ *–S*_1_	*–Cc*_1_*+S*_1_*–hL*_1_, *R*_1_–β*C*_*g*1_*–S*_1_*- h*(*kL*_1_+M_1_)
Not implementing the mechanism(1*-x*)	*–F*_1_*-gL*_1_, *R*_1_–*C*_*g*1_+*F*_1_*–kgL*_1_	*–L*_1_,*-*β*Cg*_1_*–kL*_1_

According to the replication dynamic equation, the benefits of implementing the entity responsibility mechanism and not implementing the enterprise entity responsibility mechanism for construction enterprises are determined as follows:


(1) $$\begin{array}{c} U_{C_{11}}=y\left(-C_{c1}+S_{1}\right)+\left(1-y\right)\left(-C_{c1}+S_{1}-hL_{1}\right)=-C_{c1}\\ \;+S_{1}+hL_{1}\left(y-1\right) \end{array}$$

(2) $$\begin{array}{c} U_{C_{12}}=y\left(-F_{1}-gL_{1}\right)+\left(1-y\right)\left(-L_{1}\right)=-yF_{1}-ygL_{1}+yL_{1}\\ \;-L_{1} \end{array}$$

The replication dynamic equations for the construction enterprises are:


(3) $$\begin{array}{c} F\left(x\right)=\frac{d_{x}}{d_{t}}=x\left(U_{C_{11}}-\bar{U_{C_{1}}}\right)=x\left(1-x\right)\left(U_{C_{11}}-U_{C_{12}}\right)\\ \;=x\left(1-x\right)[y\left(hL_{1}+F_{1}+gL_{1}-L_{1}\right)+S_{1}-C_{c1}+L_{1}\\ \;-hL_{1}] \end{array}$$

The benefits of strict and ordinary supervision by the government are determined as follows:


(4) $$\begin{array}{c} U_{G_{11}}=x\left(R_{1}-C_{g1}-S_{1}\right)+\left(1-x\right)\left(R_{1}-C_{g1}+F_{1}-kgL_{1}\right)\\ \;=x\left(kgL_{1}-F_{1}-S_{1}\right)+R_{1}-C_{g1}+F_{1}-kgL_{1} \end{array}$$

(5) $$\begin{array}{c} U_{G_{12}}=x\left(R_{1}-\beta C_{g1}-S_{1}-khL_{1}-hM_{1}\right)+\left(1-x\right)\\ \;\left(-\beta C_{g1}-kL_{1}\right)\\ \;=x\left(R_{1}-S_{1}-khL_{1}+kL_{1}-hM_{1}\right)-\beta C_{g1}-kL_{1} \end{array}$$

The government will constantly learn and adjust its strategies based on changes in benefits, resulting in vibrations in strategic choices. The replication dynamic equations for the government are as follows:


(6) $$\begin{array}{c} F\left(y\right)=\frac{d_{y}}{d_{t}}=y\left(U_{G_{11}}-\bar{U_{G_{1}}}\right)\\ \;=y\left(1-y\right)\left(U_{G_{11}}-U_{G_{12}}\right)\\ \;=y\left(1-y\right)\{x\left[kL_{1}\left(g+h-1\right)-F_{1}+hM_{1}-R_{1}\right]\\ \;+R_{1}+\left(\beta -1\right)C_{g1}+\left(1-g\right)kL_{1}+F_{1}\} \end{array}$$

For dynamic equations (3) and (6), let *F*(*x*) = 0 and *F*(*y*) = 0; Concurrently, we can discuss the evolutionary stability strategy of the system.

The equilibrium points existing in the replication dynamic equation system are as follows:

There are four fixed equilibrium points in the system: (0,0), (0,1), (1,0), and (1,1). There is another equilibrium point (x*, y*), where $x^{*}=\frac{R_{1}+\left(\beta -1\right)C_{g1}+\left(1-g\right)kL_{1}+F_{1}}{R_{1}+F_{1}-hM_{1}-kL_{1}\left(h+g-1\right)}$ and $y^{*}=\frac{C_{c1}+\left(h-1\right)L_{1}-S_{1}}{\left(h+g-1\right)L_{1}+F_{1}}$, when Eq. (7) are satisfied.


(7) $$\begin{array}{c} 0\leq \frac{C_{c1}+\left(h-1\right)L_{1}-S_{1}}{\left(h+g-1\right)L_{1}+F_{1}}\leq 1\\ 0\leq \frac{R_{1}+F_{1}+\left(\beta -1\right)C_{g1}+\left(1-g\right)kL_{1}}{R_{1}+F_{1}-hM_{1}-kL_{1}\left(h+g-1\right)}\leq 1 \end{array}\}$$

***Step 3.*** *Evolutionary stability strategy*

According to the method proposed by Friedman, the evolutionary stable strategy (ESS) of the differential equation system can be obtained from the local stability analysis of the Jacobian matrix *J* of the system, that is, if the Determinant *J* (*DetJ*) > 0 and Trace *J* (*TrJ*) < 0, the point is locally stable. Equations (3) and (6) constitute the system of equations whose Jacobian matrix is


(8) $$\begin{array}{c} J=[\begin{array}{c} \left(1-2x\right)\left[y\left(hL_{1}+F_{1}+gL_{1}-L_{1}\right)+S_{1}-C_{c1}+L_{1}-hL_{1}\right]\\ y\left(1-y\right)\left[kL_{1}\left(g+h-1\right)-F_{1}+hM_{1}-R_{1}\right] \end{array}\\ \begin{array}{c} x\left(1-x\right)\left(hL_{1}+F_{1}+gL_{1}-L_{1}\right)\\ \left(1-2y\right)\left\{\begin{array}{c} x\left[kL_{1}\left(g+h-1\right)-F_{1}+hM_{1}-R_{1}\right]\\ +R_{1}+\left(\beta -1\right)C_{g1}+\left(1-g\right)kL_{1}+F_{1} \end{array}\right\} \end{array}] \end{array}$$

*Det J* and *Tr J* calculation formulas for each equilibrium point in scenario 1 are listed in Table 2. Given that *h + g* = 1 is fixed, and the rewards and reputation gains from strict government supervision *R*_1_ are greater than the reputation losses in the event of an accident *M*_1_, therefore, *R*_1_ *+* (β–1) *C*_*g*1_ *+ F*_1_ *+* (1*–g*) *kL*_1_ > (β–1) *C_*g1*_ + khL*_1_ *+ hM*_1_, *S*_1_ *+ F*_1_ *+ gL*_1_ *> S*_1_ *+ L*_1_*–hL*_1_.

**TABLE 2:** The formula of the determinant for each equilibrium point.

Equilibrium point	DetJ	TrJ
(0,0)	(*S*_1_*+L*_1_*–hL*_1_*–C_*c*_*_1_).[*R*_1_*+*(β–1)*C*_*g*1_*+F*_1_*+*(1*–g*)*kL*_1_]	(*S*_1_*+L*_1_*–hL*_1_*–C_*c*_*_1_)+[*R*_1_*+*(β–1)*C*_*g*1_*+F*_1_*+*(1*–g*)*kL*_1_]
(0,1)	(*S*_1_*–C_*c*_*_1_*_+_F*_1_*_+_gL*_1_)[(1–β)*C*_*g*1_*–R*_1_*–F*_1_*-*(1*–g*)*kL*_1_]	(*S*_1_*–C_*c*_*_1_*_+_F*_1_*_+_gL*_1_)*+*[(1–β)*C*_*g*1_*–R*_1_*–F*_1_*-*(1*–g*)*kL*_1_]
(1,0)	(*C*_*c*1_–*S*_1_*–L*_1_*+hL*_1_).[(β–1) *C*_*g*1_*+khL*_1_*+hM*_1_]	(*C*_*c*1_–*S*_1_*-L*_1_*+hL*_1_)+[(β–1) *C*_*g*1_*+khL*_1_*+hM*_1_]
(1,1)	(*C*_*c*1_–*S*_1_*–F*_1_*–gL*_1_).[(1–β) *C*_*g*1_*–khL*_1_*–hM*_1_]	(*C*_*c*1_–*S*_1_*–F*_1_*–gL*_1_)+[(1–β) *C*_*g*1_*–khL*_1_*–hM*_1_]

There are nine different evolutionary stability strategies in different initial states, which are extracted in Supplementary Appendix 1. Various equilibrium scenarios are analyzed below.

(1) When the factors satisfy *C*_*c*1_ *< S*_1_ *+ L*_1_*–hL*_1_ *< S*_1_ *+ F*_1_ *+ gL*_1_, and (β–1) *C*_*g*1_ *+ khL*_1_ *+ hM*_1_ > 0, the equilibrium point is (1,1), which means that regardless of the government’s strategy, the subsidy and income saved by preventing accidents and punishment are greater than the cost of implementing the entity responsibility mechanism, and construction enterprises will choose to implement the mechanism based on income. In addition, regardless of the strategy of construction enterprises, the cost of strict supervision by the government is less than the cost of accidents, and the government chooses a strict supervision strategy.

(2) When the factors satisfy *C*_*c*1_ *< S*_1_ *+ L*_1_*–hL*_1_ *< S*_1_ *+ F*_1_ *+ gL*_1_, and *R*_1_ *+* (β–1) *Cg*_1_*+ F*_1_ *+* (1*–g*) *kL*_1_ < 0, the equilibrium point is (1,0). That is, regardless of the government’s strategy, the economic benefits are greater than the cost of implementing the entity responsibility mechanism by the construction enterprises. Therefore, construction enterprises have chosen to implement this mechanism. In addition, regardless of the strategy of construction enterprises, enforcement of stricter supervision by the government will increase the supervision cost. Therefore, the government has adopted an ordinary supervision strategy.

(3) When *C*_*c*1_ *< S*_1_ *+ L*_1_*–hL*_1_ *< S*_1_ *+ F*_1_ *+ gL*_1_, and (β–1) *C*_*g*1_ *+ khL*_1_ *+ hM*_1_ < 0 < (β–1) *Cg*_1_ *+ R*_1_ *+ F*_1_ *+* (1*–g*) *kL*_1_, the equilibrium point is (1,0), that is, whatever the government chooses, the cost paid by the construction for implementing the entity responsibility mechanism is less than the performance delivered by the mechanism; therefore, construction enterprises will implement the entity responsibility mechanism. The benefit from the government is affected by the construction enterprises, that is strict government supervision is less beneficial than that of the ordinary supervision adopted by construction enterprises implementing the entity responsibility mechanism. Thus, governments tend to choose ordinary supervision.

(4) When *S*_1_ *+ L*_1_*–hL*_1_ < *S*_1_ *+ F*_1_ *+ gL*_1_ < *C*_*c*1_, and (1–β) *C*_*g*1_ *< khL*_1_ *+ hM*_1_, the equilibrium point is (0,1), meaning, regardless of the government’s strategy, the cost paid by construction enterprises is greater than the income from implementing the entity responsibility mechanism. Therefore, from an income perspective, the construction enterprises will choose not to implement the mechanism. In addition, regardless of the strategy adopted by construction enterprises, the cost of strict supervision by the government is less than the cost of accidents and consequently, the government chooses a strict supervision strategy.

(5) When *S*_1_ *+ L*_1_*–hL*_1_ < *S*_1_ *+ F*_1_ *+ gL*_1_ < *C*_*c*1_ and (1–β) *C*_*g*1_ *> R*_1_ *+ F*_1_ *+* (1*–g*) *kL*_1_, the equilibrium point is (0,0), meaning, regardless of the government’s strategy, the economic benefits are less than the cost of implementing the entity responsibility mechanism by construction enterprises, and therefore construction enterprises choose not to implement the mechanism based on income. In addition, regardless of the construction enterprises’ strategy, the overall benefit obtained from ordinary supervision is greater than that obtained using strict supervision by the government; therefore, it is disadvantageous to incentivize the government to take strict supervision.

(6) When *S*_1_ *+ L*_1_*–hL*_1_ < *S*_1_ *+ F*_1_ *+ gL*_1_ < *C*_*c*1_, and (β–1) *C*_*g*1_ *+ khL*_1_ *+ hM*_1_ < 0 < *R*_1_ *+ F*_1_ *+* (β–1) *Cg*_1_ *+* (1*–g*) *kL*_1_, the equilibrium point is (0,1). That is, regardless of the government’s choice, construction enterprises will not implement an entity responsibility mechanism. The benefits of the government are affected by the construction enterprises, and the cost saved through ordinary government supervision is less than the strict supervision performance when construction enterprises are not implementing the entity responsibility mechanism. Therefore, the government tends to choose strict supervision.

(7) When the factors satisfy the conditions *S*_1_ *+ L*_1_*–hL*_1_ < *C*_*c*1_ < *S*_1_ *+ F*_1_ *+ gL*_1_ and (1–β) *C*_*g*1_ *< khL*_1_ *+ hM*_1_, the equilibrium point is (1,1). Therefore, regardless of the strategy adopted by construction enterprises, the benefit of strict supervision by the government is greater than that of ordinary supervision. The construction enterprises benefits are affected by the government. when the government chooses strict supervision, not implementing the mechanism is less beneficial than implementing the mechanism. Thus, construction enterprises are inclined to implement the mechanism strategy.

(8) When the factors satisfy *S*_1_ *+ L*_1_*–hL*_1_ < *C*_*c*1_ < *S*_1_ *+ F*_1_ *+ gL*_1_ and (1–β) *C*_*g*1_ > *R*_1_ *+ F*_1_ *+* (1*–g*) *kL*_1_, the equilibrium point is (0,0). meaning, regardless of the choice made by construction enterprises, the government will choose ordinary supervision. The construction enterprises benefits are affected by the government; therefore, construction enterprises will not implement the entity responsibility mechanism when the government adopts ordinary supervision.

(9) When the factors satisfy the condition *S*_1_ *+ L*_1_*–hL*_1_ < *C*_*c*1_ < *S*_1_ *+ F*_1_ *+ gL*_1_ and (β–1) *C*_*g*1_ *+ khL*_1_ *+ hM*_1_ < 0 < (β–1) *Cg*_1_ *+ R*_1_ *+ F*_1_ *+* (1*–g*) *kL*_1_, the replicated dynamic equation is asymptotically stable at point (x*, y*), the decision-making of both parties influences each other, and the decisions of the two participants depend on the change in the threshold, which is related to factors *S*_1_, *L*_1_, *C*_*C*1_, and *F*_1_.

#### Scenario 2: The Third-Party Participation Mechanism

***Step 1***. *Benefit matrix of the evolutionary game*

Third-party institutions are supervision and management institutions entrusted by the government or contractors to provide professional, fair, and objective safety supervision services in a compensatory manner. The government’s supervision cost is *C*_*g*2_. In addition, construction enterprises employ third-party institutions to solve safety management problems, which increases the safety investment cost of construction enterprises, but reduces the probability of production safety accidents; the cost of safety management will be *C*_*c*2_ (*C*_*c*2_ *> C_*c*_*_1_)*.* The definitions of the other related parameters are the same as those in Scenario 1. A payoff matrix between the government and construction enterprises under the third-party participation mechanism was established, as shown in Table 3. Third-party supervision institutions are more professional in safety supervision. Therefore, when the government chooses ordinary supervision and enterprises implement the mechanism, which can avoid the problems of slackness and insufficient resources in the process of enterprises supervision, the probability of accidents decreases from *h* in scenario 1 to *h*(1-α). α in *h*(1-α) represents the supervision effects of the third-party.

***Step 2.*** *Evolutionary game analysis of the replicator dynamics equation*

**TABLE 3:** The benefit matrix between government and construction enterprises.

Construction enterprises	Government	
	Strict supervision(*y*)	Ordinary supervision(1-*year*)
Implementing the mechanism(*x*)	*–C_*c*_*_2_*+S*_2_,*R_2_–C_*g*_*_2_*–S*_2_	*–C_*c*_*_2_*+S_2_–h*(1*–α*)*L*_2_, *R*_2_–β*C*_*g*2_*–S*_2_*–h*(1*–α*)(*kL*_2_+*M*_2_)
Not implementing the mechanism(1*-x*)	*–F*_2_*–gL*_2_, *R*_2_*–Cg*_2_*+F*_2_*–kgL*_2_	*–L*_2_, –β*Cg*_2_*–kL*_2_

The replication dynamic equation for the construction enterprises is:


(9) $$\begin{array}{c} F\left(x\right)=\frac{d_{x}}{d_{t}}=x\left(U_{C_{21}}-\bar{U_{C_{2}}}\right)=x\left(1-x\right)\left(U_{C_{21}}-U_{C_{22}}\right)\\ \;=x\left(1-x\right)\{y\left[F_{2}+h\left(1-\alpha \right)L_{2}-\left(1-g\right)L_{2}\right]\\ \;+S_{2}-C_{c2}-h\left(1-\alpha \right)L_{2}+L_{2}\} \end{array}$$

For the government, the benefits of implementing the third-party participation mechanism and not implementing the mechanism are determined as follows:

The replication dynamic equation for the government is:


(10) $$\begin{array}{c} F\left(y\right)=\frac{d_{y}}{d_{t}}=y\left(U_{G_{21}}-\bar{U_{G_{2}}}\right)=y\left(1-y\right)\left(U_{G_{21}}-U_{G_{22}}\right)\\ \;=y\left(1-y\right)\{x\left[kL_{2}\left(g-1\right)+h\left(1-\alpha \right)\left(kL_{2}+M_{2}\right)-R_{2}-F_{2}\right]\\ \;+R_{2}+\left(\beta -1\right)C_{g2}+F_{2}+kL_{2}\left(1-g\right)\} \end{array}$$

For the dynamic Eqs (9) and (10), let *F*(*x*) = 0 and *F*(*y*) = 0. Concurrently, we can discuss the evolutionary stability strategy of the system.

The equilibrium points in the replication dynamic equation system in scenario 2 are as follows:

There are four fixed equilibrium points in the system: (0,0), (0,1), (1,0), and (1,1). There is another equilibrium point (x*,y*), where $x^{*}=\frac{R_{2}+F_{2}+\left(\beta -1\right)C_{g2}+kL_{2}\left(1-g\right)}{R_{2}+F_{2}+kL_{2}\left(1-g\right)-\left(kL_{2}+M_{2}\right)h\left(1-\alpha \right)}$ and $y^{*}=\frac{S_{2}-C_{c2}-h\left(1-\alpha \right)L_{2}+L_{2}}{\left(1-g\right)L_{2}-h\left(1-\alpha \right)L_{2}-F_{2}}$, when $0\leq \frac{R_{2}+\left(\beta -1\right)C_{g2}+F_{2}+kL_{2}\left(1-g\right)}{R_{2}+F_{2}+kL_{2}\left(1-g\right)-\left(kL_{2}+M_{2}\right)h\left(1-\alpha \right)}\leq 1$, and $0\leq \frac{S_{2}-C_{c2}-h\left(1-\alpha \right)L_{2}+L_{2}}{\left(1-g\right)L_{2}-h\left(1-\alpha \right)L_{2}-F_{2}}\leq 1$.

***Step 3.*** *Evolutionary stability strategy*

Equations (9) and (10) constitute the system of the equations, and the Jacobian matrix in scenario 2 is as follows:


(11) $$\begin{array}{c} J=[\begin{array}{ccc} \left(1-2x\right)\left\{\begin{array}{c} y\left[h\left(1-\alpha \right)L_{2}-\left(1-g\right)L_{2}+F_{2}\right]\\ +S_{2}-C_{c2}-h\left(1-\alpha \right)L_{2}+L_{2} \end{array}\right\}\; & & \\ y\left(1-y\right)\left[\begin{array}{c} kL_{2}\left(g-1\right)+h\left(1-\alpha \right)\\ \left(kL_{2}+M_{2}\right)-R_{2}-F_{2} \end{array}\right]\; & & \end{array}\\ \begin{array}{ccc} x\left(1-x\right)\left[h\left(1-\alpha \right)L_{2}-\left(1-g\right)L_{2}+F_{2}\right]\; & & \\ \left(1-2y\right)\left\{\begin{array}{c} x[kL_{2}\left(g-1\right)-F_{2}-R_{2}+h\left(1-\alpha \right)\\ \left(kL_{2}+M_{2}\right)]+R{}_{2}+(\beta -1)C{}_{g2}+F{}_{2}\\ +kL_{2}\left(1-g\right) \end{array}\right\}\; & & \end{array}] \end{array}$$

*Det J* and *Tr J* calculation formulas for each equilibrium point in scenario 2 are listed in Table 4. It is assumed that *R*_2_ > *M*_2_, and thus (β–1) *Cg*_2_ + *R*_2_ + *F*_2_ + *kL*_2_ (1*–g*) > (β–1) *Cg*_2_ + *h* (1*–α*) (*kL*_2_ + *M*_2_).

**TABLE 4:** The formula of the determinant for each equilibrium point.

Equilibrium point	Det J	Tr J
(0,0)	[*S*_2_+*L*_2_–*h*(1*-α*)*L*_2_–*C*_*c*2_][*R*_2_+(β–1)*C*_*g*2_+*F*_2_+*kL*_2_(1–g)]	[*S*_2_+*L*_2_–*h*(1*–α*)*L*_2_–*C*_*c*2_]+[*R*_2_+*F*_2_+(β–1)*C*_*g*2_ +*kL*_2_(1–g)]
(1,0)	[*C*_*c*2_–*S*_2_–*L*_2_+*h*(1*–α*)*L*_2_][(β–1)*C*_*g*2_+*h*(1*–α*)(*kL*_2_+*M*_2_)]	[*C*_*c2*_–*S*_2_–*L*_2_+*h*(1–α)*L*_2_]+[(β–1)*C*_*g*2_+*h*(1*–α*)(*kL*_2_+*M*_2_]
(0,1)	(*S*_2_+*F*_2_+g*L*_2_–*C*_*c*2_) [(1–β)*C*_*g*2_–*R*_2_–*F*_2_–*k*(1*–g*)*L*_2_]	(*S*_2_+*F*_2_+g*L*_2_–*C*_*c*2_)+[-*R*_2_–*F*_2_+(1–β)*C*_*g*2_–*k*(1*–g*)*L*_2_]
(1,1)	(*C*_*c*2_–*S*_2_–*F*_2_–g*L*_2_) [(1–β)*C*_*g*2_–*h*(1*–α*) (*kL*_2_+*M*_2_)]	(*C*_*c*2_–*S*_2_–*F*_2_–g*L*_2_)+[(1–β)*C*_*g*2_–*h*(1*–α*)(*kL*_2_+*M*_2_)]

We analyze various equilibrium scenarios below, and there are 11 different evolutionary stability strategies in different initial states, which are summarized in Supplementary Appendix 2. For the sake of explaining, we simplified the 11 scenarios into seven scenarios.

(1) For construction enterprises, when the factors satisfy *Cc*_2_ > *S*_2_ + *F*_2_ + g*L*_2_ and *Cc*_2_ > *S*_2_ *+* (1*–h + hα*) *L*_2_, that is, regardless of the government’s choice, the economic benefit for the construction enterprises implementing the third-party participation mechanism is less than that of not implementing the mechanism. Therefore, construction enterprises do not implement a third participation mechanism.

(2) When the factors satisfy *Cc*_2_ < *S*_2_ *+* (1*–h + hα*) *L*_2_ and *Cc*_2_ < *S*_2_ + *F*_2_ + g*L*_2_, that is, regardless of the government’s choice, the cost paid by the construction for implementing the third-party participation mechanism is less than the performance delivered through the mechanism. Therefore, construction enterprises will adopt the strategy of implementing the mechanism.

(3) When *S*_2_ + *F*_2_ + *gL*_2_ < *Cc*_2_ < *S*_2_ *+* (1*–h + hα*) *L*_2_ or *S*_2_ *+* (1*–h + hα*) *L*_2_ < *Cc*_2_ < *S*_2_ + *F*_2_ + g*L*_2_, the benefits of construction enterprises are affected by the government. When *S*_2_ + *F*_2_ + g*L*_2_ < *Cc*_2_ < *S*_2_ *+* (1*–h + hα*) *L*_2_ and *R*_2_ + (β–1) *C*_*g*2_ + *F*_2_ + *kL*_2_ (1*–g*) < 0, enterprises will implement the mechanism, and the equilibrium point is (1,0). When *S*_2_ + *F*_2_ + g*L*_2_ < *Cc*_2_ < *S*_2_ *+* (1*–h + hα*) *L*_2_ and (β–1) *C*_*g*2_ + *h* (1*–α*) (*kL*_2_ *+ M*_2_) > 0, construction enterprises will not implement the mechanism, and the equilibrium point is (0,1). When *S*_2_ + (1–*h*+ *hα*) *L*_2_ < *Cc*_2_ < *S*_2_ + *F*_2_ + g*L*_2_ and the government’s decision making remains unchanged, the enterprise’s decision making is the reverse.

(4) For the government, when the factors satisfy the condition *R*_2_ + (β–1) *C*_*g*2_ + *F*_2_ + *kL*_2_ (1*–g*) < 0, regardless of the strategy adopted by construction enterprises, the cost saved by ordinary government supervision is greater than the performance delivered through strict supervision; therefore, the government adopts an ordinary supervision strategy.

(5) When the factors satisfy the condition (β–1) *C*_*g*2_ + *h* (1*–α*) (*kL*_2_ *+ M*_2_) > 0, it means that regardless of the construction enterprises’ strategy, the cost of strict supervision by the government is less than the cost of accidents, and thus the government chooses a strict supervision strategy.

(6) When (β–1) *C*_*g*2_ + *h* (1*–α*) (*kL*_2_ *+ M*_2_) < 0 *< R*_2_ + (β–1) *C*_*g*2_ + *F*_2_ + *kL*_2_ (1*–g*), government’s strategy is determined by the decision making of construction enterprises. When *C*_*c*2_ *> S*_2_ + *F*_2_ + g*L*_2_ and *C*_*c*2_ *> S*_2_ + (1*–h + hα*) *L*_2_, the government will choose strict supervision; when *C*_*c*2_ *< S*_2_ + *F*_2_ + g*L*_2_ and *C*_*c*2_ *< S*_2_+ (1*–h + hα) L*_2_, the government will choose ordinary supervision.

(7) When *S*_2_ *+* (1*–h + hα*) *L*_2_ *> C_*c*_*_2_ *> S_2_ + F*_2_ *+ gL*_2_ and (β–1) *C*_*g*2_ + *h* (1*–α*) (*kL*_2_ *+ M*_2_) < 0 *< R*_2_ + (β–1) *C*_*g*2_ + *F*_2_ + *kL*_2_ (1*–g*), the decision-making of the two participants in the game cannot be determined, and the strategies of both sides influence each other. In this case, the equilibrium points are (1,0), (0,1) and (x*, y*). The equilibrium points depend on the changes in x* and y*, which are related to the factors *S*_2_, *L*_2_, *C*_*C*2_, and *F*_2_.

## Simulation and Analysis

To describe the stability of behavioral strategies more intuitively, this section uses numerical simulation to analyze the factors influencing the implementation of the two supervision mechanisms by enterprises in different scenarios, and validates the effectiveness of the two supervision mechanisms in improving supervision efficiency, which helps motivate construction enterprises to introduce appropriate safety supervision mechanisms.

### Results and Analysis for Scenario 1

To better reflect the actual situation of the project construction stage, this study drew lessons from some references and investigated some construction projects, and the necessary data and materials were collected, as shown in Table 5. Both the initial probabilities *x* and *y* of the government and construction enterprises adopting the positive supervision strategy were assumed to be 0.5 in the simulation. Moreover, the effectiveness of improving supervision efficiency and the influencing factors of implementing the enterprise entity responsibility mechanism were analyzed.

**TABLE 5:** The parameter values.

Parameter	*C*_*g*1_	*R*_1_	*M*_1_	*C*_*c*1_	*S*_1_	*F*_1_	*L*_1_	*g*	*h*	β	*k*
**Values**	0.5	1.5	1.2	1	0.5	0.7	1.5	0.6	0.4	0.5	1

**FIGURE 2:**
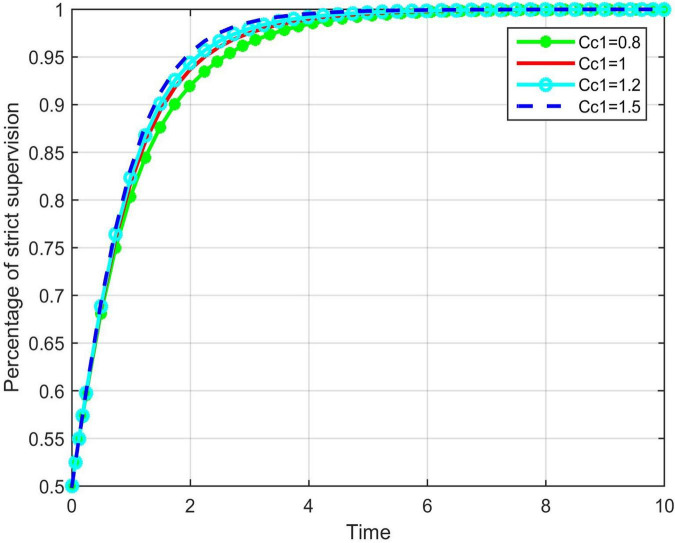
The impact of *C*_*c*1_ on the evolution trend of government strict super.

To verify the effectiveness of the implementation of enterprise entity responsibility mechanisms in improving the supervision efficiency, it was assumed that the implementation of the mechanism of construction enterprises is reflected by the value of *C*_*c*1_; the larger the value of *C*_*c*1_, the more effective is the mechanism of the enterprises. Based on the values of the supervision cost, *C*_*c*1_ was set to 0.8, 1, 1.2, and 1.5, respectively. The MATLAB software was used for the numerical simulation analysis of the evolutionary game process of the government, as shown in Figure 2.

As shown in the figure above, as the value of *C*_*c*1_ continues to increase, the time t taken for the system to stabilize decreases; that is, the greater the degree of implementation of the main responsibility mechanism of the enterprise is, the faster the game system stabilizes. At this point, strict government supervision can achieve the maximum benefit, indicating that the implementation of the mechanism by construction enterprises has a positive effect on optimizing the government’s safety supervision.

**FIGURE 3:**
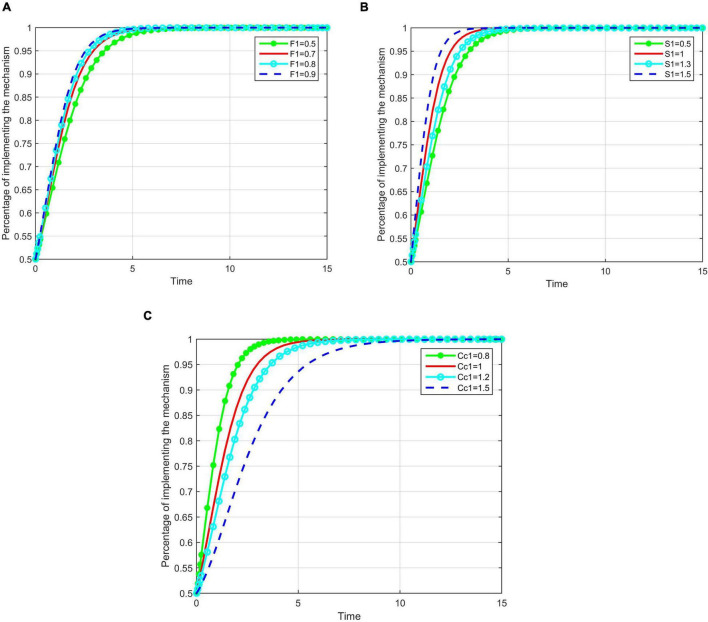
The impact of fine, subsidy and cost on implementing the first mechanism. **(A)** The impact of fine on implementing the first mechanism. **(B)** The impact of subsidy on implementing the first mechanism. **(C)** The impact of cost on implementing the first mechanism.

With other factors constant, we studied the impact of *F*_1_, *S*_1_, and *C*_*c*1_ on enterprises that implemented enterprise entity responsibility mechanisms. *F*_1_ was set as 0.5, 0.7, 0.8, 0.9; *S*_1_ was set as 0.5; 1, 1.3, 1.5. and *C*_*c*1_ was set as 0.8, 1, 1.2, 1.5. A numerical simulation analysis of the evolutionary game process is shown in Figure 3.

As shown in Figure 3, the values of *F*_1_, *S*_1_, and *C*_*c*1_ have an impact on enterprises’ strategies. When other factors remain constant, with the increase in *F*_1_, the rate of construction enterprises that tend to use the enterprise entity responsibility mechanism increases, and the time *t* taken for the system to stabilize becomes shorter. The only change is the speed at which the steady state is reached; the final steady state will remain constant. When *F*_1_ reaches a certain level, increasing penalties have little impact on promoting enterprises to implement the enterprise entity responsibility mechanism. However, the time *t* taken for the system to stabilize does not decrease with increasing *S*_1_. Therefore, the continuous increase of *S*_1_ will not promote the implementation of the mechanism; therefore, *S*_1_ should be kept at an appropriate level. As *C*_*c*1_ increases, the time taken for enterprises to stabilize increases, indicating that the cost spent by enterprises inhibits the introduction of the mechanism.

### Results and Analysis for Scenario 2

The data in Scenario 2 were summarized and averaged, as shown in Table 6. Both the initial probabilities *x* and *y* of the government and construction enterprises adopting the positive supervision strategy were assumed to be 0.5. The effectiveness of improving the supervision efficiency and the influencing factors of implementing the third-party mechanism were analyzed.

**TABLE 6:** The parameter values.

Parameter	*C*_*g*2_	*R*_2_	*M*_2_	*C*_*c*2_	*S*_2_	*F*_2_	*L*_2_	*g*	*h*	β	*k*
**Values**	0.5	1.8	1	1.5	0.8	0.5	1	0.6	0.4	0.5	1

**FIGURE 4:**
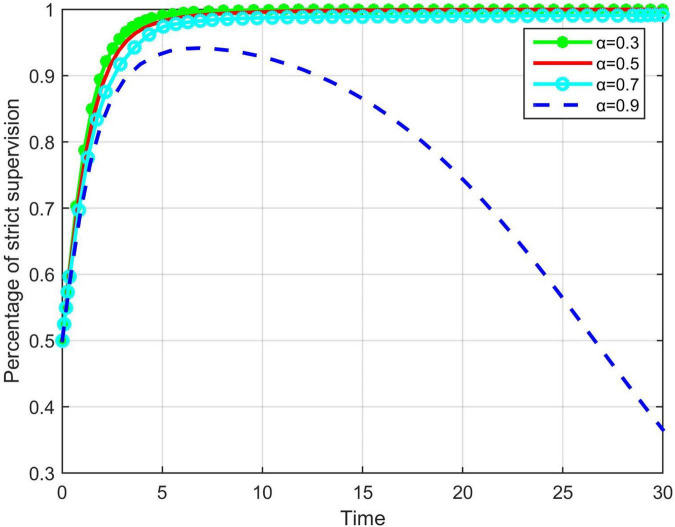
The impact of *a* on the evolution trend of government strict supervision.

Considering that the third-party supervision institutions do not always supervise the construction enterprises, nor can they always find unsafe behaviors of front-line workers, α is used in this model to represent the probability that the third-party supervision institutions discover and correct unsafe behaviors of enterprises; 0 < α < 1, the larger the value of α, the more effective the third-party participation mechanism of the enterprise is. The impact of the mechanism on the evolutionary results of government will be discussed. In the numerical simulation, we set α as 0.3, 0.5, 0.7, 0.9. A numerical simulation analysis of the evolutionary game process is shown in Figure 4.

As shown in Figure 4, as the value of α continues to increase, the time *t* taken for the system to reach a stable state of strict supervision also increases; that is, the greater the degree of implementation of the third-party participation mechanism of the enterprises, the slower the game system reaches a stable state. This shows that the relationship of the third-party supervision with government supervision is that of a substitute; the greater the supervision intensity, the smaller the supervision intensity of the government. α = 0.9 means that the intensity of third-party supervision is so great that the government can change the existing supervision intensity. Without considering the damage to the government’s reputation, the government will choose ordinary supervision because the third-party supervision replaces their supervision responsibilities.

**FIGURE 5:**
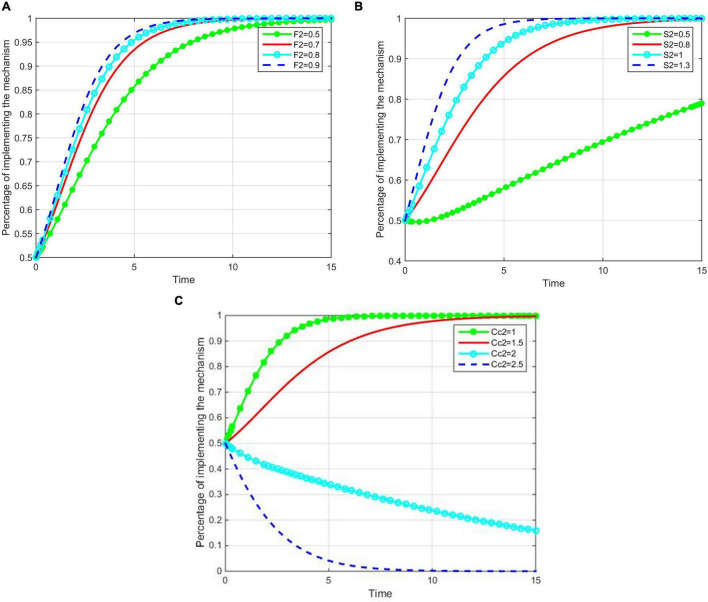
The impact of fine, subsidy and cost on implementing the second mechanism. **(A)** The impact of fine on implementing the second mechanism. **(B)** The impact of subsidy on implementing the second mechanism. **(C)** The impact of cost on implementing the second mechanism.

The impact of penalties *F*_2_, subsidies *S*_2_, and the cost *C*_*c*2_ on the evolutionary results of construction enterprises are discussed below. The value of *F*_2_ was set as 0.5, 0.7, 0.8, 0.9; *S*_2_ was set as 0.5, 0.8, 1, and 1.3; and *C*_*c*2_ was set as 1, 1.5, 2, 2.5. A numerical simulation analysis of the evolutionary game process is shown in Figure 5.

As shown in Figure 5, the values of *F*_2_, *S*_2_, and *C*_*c*2_ affect the construction enterprises’ strategy. With a continuous increase in *F*_2_, the time *t* for the system to reach the stable state decreases, which indicates that the greater the penalty, the more the construction enterprises tend to choose the third-party participation mechanism. Figure 5 shows that the probability of introducing third-party participation is sensitive to the subsidies *S*_2_ and the cost *C*_*c*2_, and an increase in government subsidies will encourage enterprises to introduce the third-party participation mechanism. When the cost is smaller than the threshold value, the probability of introducing the third-party participation mechanism converges to one. When the cost is greater than the threshold value, the probability converges to zero, and the convergence speed decreases with the decrease in cost. This indicates that the implementation of the mechanisms is most sensitive to the cost *C*_*c*2_, which restrains the enterprises from introducing a third-party participation mechanism.

## Discussion

### Main Findings

Based on the analysis in this study, different equilibrium points were obtained for the two scenarios. Through numerical simulations, we validated and compared the effectiveness of the two mechanisms in improving the supervision efficiency and discussed the factors influencing the ideal equilibrium conditions in each scenario. The main findings of this study are as follows:

The enterprise entity mechanism is an effective approach for promoting the efficiency of government supervision; by introducing this mechanism, enterprises can share the responsibility of government supervision while minimizing the probability of accidents, reducing the government cost of sharing accident risks, and maximizing the government’s interests. Therefore, construction enterprises should improve their security crisis awareness and strengthen their internal management to achieve self-improvement. The decision by the enterprises to introduce the mechanism is mainly affected by the cost spent by the enterprises, which restrains the introduction of the mechanism. Concurrently, subsidies and penalties obtained by enterprises also have an impact on whether or not the mechanism is introduced. However, when the mechanism reaches a certain level, the effect of penalties is not obvious, and more subsidies are not always working better, which gives rise to the need for the government formulating policies to ensure that both penalties and subsidies reach the appropriate level.

The third-party participation mechanism has a positive effect on government supervision and can promote ordinary government supervision. In this condition, the government can accomplish its intended regulatory purposes with fewer resources, which improves the efficiency of government supervision. The effects of multiple managerial factors on the introduction of the mechanism, including punishment cost, subsidies, and supervision cost, are examined. The subsidies and cost for construction enterprises are the decisive factors that decide whether or not the enterprises undertake the mechanism. Enterprises are less sensitive to fines relative to the subsidies and costs. On the one hand, as the introduction of the mechanism is a measure and management requiring high investment costs, whether it is invested or not often has a strong inverse relationship with the profit of a project. On the other hand, compared with the enterprise entity responsibility mechanism, the punishment of enterprises not introducing the third-party participation mechanism is lower. As long as there is no accident in the project, no matter how big the danger is, it is difficult to effectively punish the enterprises. Therefore, enterprises are willing to take risks with a fluke mentality for the sake of profit. Therefore, it is suggested to (1) give full control to the multiparty synergistic effect of third-party institutions, enterprises, institutions, and research institutes; (2) innovate the cooperative governance mechanism; and (3) construct an effective mechanism of joint supervision and collaborative governance. Moreover, the government should increase subsidies to strengthen the introduction of third-party regulatory mechanisms for companies.

The third-party participation mechanism is a faster way to ensure that the system reaches a stable state and plays the most significant role in government supervision. This phenomenon aligns with actual situations. On the one hand, the third-party participation can attract professional and technical personnel engaged in quality and safety supervision to give full control to these professional and technical personnel. On the other hand, the participation of third-party institutions can provide correct and effective institutional information for the government by virtue of their working environment advantages and compensate for the information asymmetry between the government supervision departments and construction enterprises.

### Theoretical Implications

First, herein, a solution is provided for the deficiencies of the existing government’s supervision mode and the construction safety supervision system in China by establishing a type of synergistic and complementary safety supervision mechanism. In this study, construction enterprises not only played the role of safety producers but also safety supervisors, and the blended supervision mechanism integrating the government’s passive supervision and enterprises’ active supervision are introduced. This mechanism was found to effectively promote government supervision by reducing the government supervision cost and accident rates. This is a pragmatic alternative to traditional government safety supervision, which enriches the theoretical system of construction safety management. The advantages of the synergistic and complementary mechanisms were given, the validity of the proposed mechanism was tested, and the analysis of the application results highlighted the advantages of the mode and provided the basis for popularization and application.

Second, this study contributes to the literature in construction safety supervision by offering a better understanding of how interactions between the government and enterprises affect the government’s strict supervision. To abstract the practical problems in a rational way, we develop stylized evolutionary game models as a simplified version of reality, which outlines the decision-making interactions of the government and construction enterprises and the implementation of enterprises’ active supervision mechanisms in different situations. The models excavate the decision logic behind the safety supervision behaviors of enterprises and government, reveal the root cause of the problems existing in the government, and explain how blended supervision achieves effective supervision outcomes. Our proposed research models undoubtedly provide a coherent framework as a first step toward improving the traditional “vertical supervision” mode. In addition to the construction safety supervision, this model can be used to demonstrate the supervision mechanisms of other industries.

Finally, as per the results, the proposed penalty–reward scenario was found to balance the interests of the government and construction enterprises; thereby, meeting the requirement of active supervision by construction enterprises. The study quantitatively analyzes the feasibility and effectiveness of enterprise entity responsibility and third-party participation and helps to deepen the understanding of the role of construction enterprises and third parties in construction safety supervision. The simulation illustrates the effectiveness and identifies the factors influencing the implementation of the two mechanisms, which will provide useful insights for the government to formulate more scientific and reasonable incentive policies and then have a certain reference value for the realization of active enterprise supervision and accident reduction goals.

### Practical Implications

First, this study provides two alternative mechanisms for the effective supervision of the government. The new mechanisms optimize the path of government supervision, strengthen the role of a third-party in the supervision of construction safety, which shares the government’s supervisory responsibility, supplement for the plight of insufficient government safety supervision force and low technical level of the hierarchical supervision. On the other hand, it also responds to national policies. Moreover, the third-party participation mechanism proposed provides enterprises with more professional engineering project safety monitoring and management services and timely supervision, urges the implementation of all parties responsible for the construction process, rectifies the problems and safety risks existing in safety management, and curbs the occurrence of safety accidents.

Second, the blended mechanism established in this study helps to restrict the insufficient safety behavior of enterprises, improve the consciousness and initiatives of enterprises which will lead to a safer working environment on construction sites, improve the efficiency of government supervision and play a vital role in reducing the occurrence of accidents. The enterprise entity responsibility mechanism proposed in this paper promotes enterprises to have a stable social environment and a good development environment which cannot only effectively reduce the economic losses caused by safety accidents, but also create a good social image for enterprises, so that enterprises can obtain social and economic benefits of the dual protection.

Finally, the analysis of the influencing factors that affect the introduction of the two mechanisms guides the government to regulate and supervise the behavior of the responsible subjects. This paper analyzes the root of the problems existing in the government supervision and actively explores the countermeasures and concrete measures to improve the government supervision of construction safety. The government can promote the implementation of enterprise supervision mechanisms by reducing supervision costs, appropriately increasing punishments, and establishing effective incentive mechanisms which balance government safety supervision and enterprise self-management and have a great significance in improving the efficiency of safety supervision and ensuring safe production.

### Limitations and Future Research

Our research comes with certain limitations. The model established in this study only considers the economic losses resulting from safety accidents. As construction safety supervision is complex, the non-economic losses caused by the social environmental losses and psychological trauma are often difficult to be quantified and thus neglected to a certain extent in this study. Therefore, future research should consider these non-economic losses.

## Conclusion

This study used an evolutionary game model to describe the decision-making interactions between the government and construction enterprises under the enterprise entity responsibility and third-party participation mechanisms. In addition, a series of simulation experiments were conducted to illustrate the factors influencing the implementation of the mechanisms. The principal conclusions of this study are as follows: First, the implementation of these two mechanisms positively affects government supervision. Second, the third-party participation mechanism has a better supervision effect than the implementation of the enterprise entity mechanism. Finally, the implementation of the two mechanisms is influenced by punishment, subsidy, and cost, and it has different sensitivities to three influencing factors that guide the government to regulate and supervise the behavior of responsible subjects. The study provides a theoretical framework for exploring the optimization mechanism of the government, which restricts enterprises’ insufficient safety behavior and improves the efficiency of government supervision.

## Data Availability Statement

The raw data supporting the conclusions of this article will be made available by the authors, without undue reservation.

## Author Contributions

All authors listed have made a substantial, direct, and intellectual contribution to the work, and approved it for publication.

## Conflict of Interest

The authors declare that the research was conducted in the absence of any commercial or financial relationships that could be construed as a potential conflict of interest.

## Publisher’s Note

All claims expressed in this article are solely those of the authors and do not necessarily represent those of their affiliated organizations, or those of the publisher, the editors and the reviewers. Any product that may be evaluated in this article, or claim that may be made by its manufacturer, is not guaranteed or endorsed by the publisher.
