# Supplementary material for: Developing a Decision-Making Model for Construction Safety Behavior Supervision: An Evolutionary Game Theory-Based Analysis
Source: Front Psychol. 2022 Apr 7;13:861828. doi: 10.3389/fpsyg.2022.861828 (PMC9021837; doi:10.3389/fpsyg.2022.861828)
Supplement: Supplementary file 1 [file Table_1.docx]

**Appendix 1** Analysis results of system stability under scenario 1

| **Scenario** | **Equilibrium point** | **DetJ** | **TrJ** | **Stability** |
| --- | --- | --- | --- | --- |
| *S*_1_*+F*_1_*+gL*_1_*>S*_1_*+L*_1_*-hL*_1_*>C_c_*_1_  (*β-*1)*C_g_*_1_*+khL*_1_*+hM*_1_*>*0 | (0,0) | + | + | Instability |
|  | (1,0) | - | Indefinite | Instability |
|  | (0,1) | - | Indefinite | Instability |
|  | (1,1) | + | - | ESS |
| *S*_1_*+F*_1_*+gL*_1_*>S*_1_*+L*_1_*-hL*_1_*>C_c_*_1_  *R*_1_*+*(*β-*1)*C_g_*_1_*+F*_1_*+*(1*-g*)*kL*_1_*<*0 | (0,0) | - | Indefinite | Instability |
|  | (1,0) | + | - | ESS |
|  | (0,1) | + | + | Instability |
|  | (1,1) | - | Indefinite | Instability |
| *S*_1_*+F*_1_*+gL*_1_*>S*_1_*+L*_1_*-hL*_1_*>C_c_*_1_  (*β-*1)*C_g_*_1_*+khL*_1_*+hM*_1_<0<*R*_1_*+*(*β-*1)*C_g_*_1_*+F*_1_*+*(1*-g*)*kL*_1_ | (0,0) | + | + | Instability |
|  | (1,0) | + | - | ESS |
|  | (0,1) | - | Indefinite | Instability |
|  | (1,1) | - | Indefinite | Instability |
| *S*_1_*+L*_1_*-hL*_1_<*S*_1_*+F*_1_*+gL*_1_<*C_c_*_1_  (*β-*1)*C_g_*_1_*+khL*_1_*+hM*_1_*>*0 | (0,0) | - | Indefinite | Instability |
|  | (1,0) | + | + | Instability |
|  | (0,1) | - | + | ESS |
|  | (1,1) | - | Indefinite | Instability |
| *S*_1_*+L*_1_*-hL*_1_<*S*_1_*+F*_1_*+gL*_1_<*C_c_*_1_  *R*_1_*+*(*β-*1)*C_g_*_1_*+F*_1_*+*(1*-g*)*kL*_1_*<*0 | (0,0) | + | - | ESS |
|  | (1,0) | - | Indefinite | Instability |
|  | (0,1) | - | Indefinite | Instability |
|  | (1,1) | + | + | Instability |
| *S*_1_*+L*_1_*-hL*_1_<*S*_1_*+F*_1_*+gL*_1_<*C_c_*_1_  (*β-*1)*C_g_*_1_*+khL*_1_*+hM*_1_<0<*R*_1_*+*(*β-*1)*C_g_*_1_*+F*_1_*+*(1*-g*)*kL*_1_ | (0,0) | - | Indefinite | Instability |
|  | (1,0) | - | Indefinite | Instability |
|  | (0,1) | + | - | ESS |
|  | (1,1) | + | + | Instability |
| *S*_1_*+L*_1_*-hL*_1_<*C_c_*_1_<*S*_1_*+F*_1_*+gL*_1_  (*β-*1)*C_g_*_1_*+khL*_1_*+hM*_1_*>*0 | (0,0) | - | Indefinite | Instability |
|  | (1,0) | + | + | Instability |
|  | (0,1) | - | Indefinite | Instability |
|  | (1,1) | + | - | ESS |
| *S*_1_*+L*_1_*-hL*_1_<*C_c_*_1_<*S*_1_*+F*_1_*+gL*_1_  *R*_1_*+*(*β-*1)*C_g_*_1_*+F*_1_*+*(1*-g*)*kL*_1_*<*0 | (0,0) | + | - | ESS |
|  | (1,0) | - | Indefinite | Instability |
|  | (0,1) | + | + | Instability |
|  | (1,1) | - | Indefinite | Instability |
| *S*_1_*+L*_1_*-hL*_1_<*C_c_*_1_<*S*_1_*+F*_1_*+gL*_1_  (*β-*1)*C_g_*_1_*+khL*_1_*+hM*_1_<0<*R*_1_*+*(*β-*1)*C_g_*_1_*+F*_1_*+*(1*-g*)*kL*_1_ | (0,0) | - | Indefinite | Instability |
|  | (1,0) | - | Indefinite | Instability |
|  | (0,1) | - | Indefinite | Instability |
|  | (1,1) | - | Indefinite | Instability |
|  | (*x^*^*,*y^*^*) | + | - | ESS |
