# Supplementary material for: Developing a Decision-Making Model for Construction Safety Behavior Supervision: An Evolutionary Game Theory-Based Analysis
Source: Front Psychol. 2022 Apr 7;13:861828. doi: 10.3389/fpsyg.2022.861828 (PMC9021837; doi:10.3389/fpsyg.2022.861828)
Supplement: Supplementary file 2 [file Table_2.docx]

**Appendix 2** Analysis results of system stability under scenario 2

| **Scenario** | **Equilibrium point** | **Det J** | **Tr J** | **Stability** |
| --- | --- | --- | --- | --- |
| *C_c_*_2_*>S*_2_+*F*_2_+g*L*_2_, *C_c_*_2_*>S*_2_*+*(1*-h+hα*)*L*_2_  *R*_2_+(*β-*1)*C_g_*_2_+*F*_2_+*kL*_2_(1*-g*)<0 | (0,0) | + | - | ESS |
|  | (1,0) | - | Indefinite | Instability |
|  | (0,1) | - | Indefinite | Instability |
|  | (1,1) | + | + | Instability |
| *S*_2_+*F*_2_+g*L*_2_*>C_c_*_2_*>S*_2_*+*(1*-h+hα*)*L*_2_  *R*_2_+(*β-*1)*C_g_*_2_+*F*_2_+*kL*_2_(1*-g*)<0 | (0,0) | + | - | ESS |
|  | (1,0) | - | Indefinite | Instability |
|  | (0,1) | + | + | Instability |
|  | (1,1) | - | Indefinite | Instability |
| *S*_2_+*F*_2_+g*L*_2_*>Cc*_2_, *S*_2_*+*(1*-h+hα*) *L*_2_>*Cc*_2_  *R*_2_+(*β-*1)*C_g_*_2_+*F*_2_+*kL*_2_(1*-g*)<0 | (0,0) | - | Indefinite | Instability |
|  | (1,0) | + | - | ESS |
|  | (0,1) | + | + | Instability |
|  | (1,1) | - | Indefinite | Instability |
| *S*_2_*+*(1*-h+hα*)*L*_2_*>C_c_*_2_*>S*_2_+*F*_2_+g*L*_2_  *R*_2_+(*β-*1)*C_g_*_2_+*F*_2_+*kL*_2_(1*-g*)<0 | (0,0) | - | Indefinite | Instability |
|  | (1,0) | + | - | ESS |
|  | (0,1) | - | Indefinite | Instability |
|  | (1,1) | + | + | Instability |
| *C_c_*_2_>*S*_2_+*F*_2_+g*L*_2_, *C_c_*_2_*>S*_2_*+*(1*-h+hɑ*)*L*_2_  (*β-*1)*C_g_*_2_+*h*(1*-α*)(*kL*_2_+*M*_2_)*>0* | (0,0) | - | Indefinite | Instability |
|  | (1,0) | + | + | Instability |
|  | (0,1) | - | + | ESS |
|  | (1,1) | - | Indefinite | Instability |
| *S*_2_+*F*_2_+g*L*_2_*>C_c_*_2_*>S*_2_*+*(1*-h+hα*)*L*_2_  (*β-*1)*C_g_*_2_+*h*(1*-α*)(*kL*_2_+*M*_2_)*>0* | (0,0) | - | Indefinite | Instability |
|  | (1,0) | + | + | Instability |
|  | (0,1) | - | Indefinite | Instability |
|  | (1,1) | + | - | ESS |
| *S*_2_+*F*_2_+g*L*_2_*>C_c_*_2_, *S*_2_*+*(1*-h+hα*)*L*_2_*>C_c_*_2_  (*β-*1)*C_g_*_2_+*h*(1*-α*)(*kL*_2_+*M*_2_)*>*0 | (0,0) | + | + | Instability |
|  | (1,0) | - | Indefinite | Instability |
|  | (0,1) | - | Indefinite | Instability |
|  | (1,1) | + | - | ESS |
| *S*_2_*+*(1*-h+hα*)*L*_2_*>C_c_*_2_*>S*_2_+*F*_2_+g*L*_2_  (*β-*1)*C_g_*_2_+*h*(1*-α*)(*kL*_2_+*M*_2_)*>*0 | (0,0) | + | + | Instability |
|  | (1,0) | - | Indefinite | Instability |
|  | (0,1) | + | - | ESS |
|  | (1,1) | - | Indefinite | Instability |
| *C_c_*_2_>*S*_2_+*F*_2_+g*L*_2_, *C_c_*_2_*>S*_2_*+*(1*-h+hα*)*L*_2_  (*β-*1)*C_g_*_2_+*h*(1*-α*)(*kL*_2_+*M*_2_)*<*0*<R*_2_+(*β-*1)*C_g_*_2_+*F*_2_+*kL*_2_(1*-g*) | (0,0) | - | Indefinite | Instability |
|  | (1,0) | - | Indefinite | Instability |
|  | (0,1) | + | - | ESS |
|  | (1,1) | + | + | Instability |
| *S*_2_+*F*_2_+g*L*_2_*>C_c_*_2_, *S*_2_*+*(1*-h+hα*)*L*_2_*>C_c_*_2_  (*β-*1)*C_g_*_2_+*h*(1*-α*)(*kL*_2_+*M*_2_)*<*0*<R*_2_+(*β-*1)*C_g_*_2_+*F*_2_+*kL*_2_(1*-g*) | (0,0) | + | + | Instability |
|  | (1,0) | + | - | ESS |
|  | (0,1) | - | Indefinite | Instability |
|  | (1,1) | - | Indefinite | Instability |
| *S*_2_*+*(1*-h+hα*)*L*_2_*>C_c2_>S*_2_+*F*_2_+g*L*_2_  (*β-*1)*C_g_*_2_+*h*(1*-α*)(*kL*_2_+*M*_2_)*<*0*<R*_2_+(*β-*1)*C_g_*_2_+*F*_2_+*kL*_2_(1*-g*) | (0,0) | + | + | Instability |
|  | (1,0) | + | - | ESS |
|  | (0,1) | + | - | ESS |
|  | (1,1) | - | Indefinite | Instability |
|  | (*x^*^*, *y^*^*) | + | - | ESS |
